# Prediction of spatiotemporal patterns of neural activity using a higher-order Markov representation of instantaneous pairwise maximum entropy model

**DOI:** 10.1186/1471-2202-12-S1-P245

**Published:** 2011-07-18

**Authors:** Mehdi Aghagolzadeh, Karim Oweiss

**Affiliations:** 1 Department of Electrical and Computer Engineering, Michigan State University, East Lansing, MI, 48823, USA; 2 Neuroscience Program, Michigan State University, East Lansing, MI, 48823, USA

## 

Maximum entropy (MaxEnt) models have been suggested to fit the spatiotemporal patterns of simultaneously observed neuronal spike trains. To build such models, the distribution, *p_x_*(*X*), needs to be determined, where *X* is a sequence of neural patterns formed from the vector *X*^1^,…,*X^n^* for *n* time samples, where *X^i^* is a sample of the spike train of a population at time *i* . Each neural pattern itself is a binary sequence, for which one represents a spike, and zero otherwise. MaxEnt models are constructed by matching the moments between neuronal constituents with the analogous empirical estimates from the distribution *p_x_*(*X*).

MaxEnt models that incorporate the first and second order interactions among neurons, also referred to as the instantaneous pairwise MaxEnt model or the Ising model, are expressed as

where the individual, *h_i_*, and coupling, *J_ij_*, terms are parameters associated with the first and second order interactions, respectively, and *Z* is a normalization factor to insure . This model was shown to be effective in representing almost 90 percent of the information in small populations (less than 20 neurons) [1,2]. The main disadvantage of this model, however, is that it ignores the temporal correlation that represents the effect of the history of spiking activity on the current population state. Extending the width of neural patterns has been proposed as a method to incorporate spiking history. It destroys, however, the information in precise spike timings [3].

To incorporate the history term without compromising information in spike times, a higher-order Markov model is constructed in which patterns from different time samples are added. An example first-order Markov representation is expressed as

where  and  are the individual, and ,  and  are the coupling terms among the neuronal elements. This spatiotemporal model is more general than the representation introduced in Marre et al [4] to account for history terms , but at the expense of increasing the number of parameters.

We evaluated the performance of the extended MaxEnt model in predicting the activity pattern of cat V1 neurons in response to drifting grating stimuli. A population of 21 neurons was recorded with 5 tetrodes. We compared the prediction power of this model with the instantaneous pairwise MaxEnt model, as well as the independent model. We show that the iterative scaling algorithm makes the extended model converge faster compared to [4], and also reduces the Kullback-Leibler divergence between the estimated and true distributions by 20 percent. Taken together, these results suggest the importance of accounting for temporal correlations in predicting the spatiotemporal patterns.

## References

[B1] Schneidman E Berry MJ Segev R Bialek W Weak pairwise correlations imply strongly correlated network states in a neural population Nature 2006 440 1012 1007 10.1038/nature04701 PMC1785327 16625187

[B2] Tang A Jackson D Hobbs J Chen W Smith JL Patel H Prieto A Petrusca D Grivich MI Sher A A maximum entropy model applied to spatial and temporal correlations from cortical networks in vitro Journal of Neuroscience 2008 28 505 10.1523/JNEUROSCI.3359-07.2008 18184793 PMC6670549

[B3] Truccolo W Hochberg LR Donoghue JP Collective dynamics in human and monkey sensorimotor cortex: predicting single neuron spikes Nature neuroscience 2009 19966837 10.1038/nn.2455 PMC2820252

[B4] Marre O Boustani El S Fregnac Y Destexhe A Prediction of spatiotemporal patterns of neural activity from pairwise correlations Physical review letters 2009 102 138101 10.1103/PhysRevLett.102.138101 19392405

